# Medical Nutrition Therapy for Women with Gestational Diabetes: Current Practice and Future Perspectives

**DOI:** 10.3390/nu17071210

**Published:** 2025-03-30

**Authors:** Louisa Cheong, Lawrence Siu-Chun Law, Li Ying Lyeann Tan, Amal Al-Amri Amal, Chin Meng Khoo, Pei Chia Eng

**Affiliations:** 1 Division of Endocrinology, Department of Medicine, National University Hospital, Singapore 119074, Singapore; louisa.cheong@mohh.com.sg (L.C.); sclaw@alumni.duke.edu (L.S.-C.L.); lyeann.tan@mohh.com.sg (L.Y.L.T.); chin_meng_khoo@nuhs.edu.sg (C.M.K.); 2 Department of Internal Medicine, Nizwa Hospital, Nizwa P.O. Box 1222, Oman; smart6doctor@gmail.com; 3 Department of Metabolism, Digestion and Reproduction, Imperial College London, London W12 0NN, UK

**Keywords:** gestational diabetes, medical nutrition therapy, carbohydrates

## Abstract

Gestational diabetes mellitus (GDM) is a complication that affects 20% of pregnancies worldwide. It is associated with adverse short- and long-term cardiometabolic outcomes for both mother and infant. Effective management of GDM involves lifestyle modifications, including medical nutrition therapy (MNT) and physical activity (PA), with the addition of insulin or metformin if glycaemic control remains inadequate. However, substantial gaps persist in the determination of optimal medical nutrition therapy (MNT) for women with GDM. Challenges in MNT include individual variation in glucose tolerance and changing maternal physiology and dietary requirements during pregnancy. Achieving optimal glycaemic control depends on careful macronutrient balance, particularly the distribution and quality of carbohydrate intake and sufficient protein and fat intake. Additionally, micronutrient deficiencies, such as inadequate vitamin D, calcium, and essential minerals, may exacerbate oxidative stress, inflammation, and glycaemic dysregulation, further impacting foetal growth and development. Cultural beliefs and dietary practices among pregnant women can also hinder adherence to recommended nutritional guidelines. Conditions like hyperemesis gravidarum (HG) affect ~1% to 2% of pregnant women can result in unintended energy and nutrient deficits. This special issue explores the current evidence and major barriers to optimising dietary therapy for women with GDM. It also identifies future research priorities to advance clinical practice, improve maternal and foetal outcomes, and address gaps in personalised nutrition interventions.

## 1. Introduction

Gestational diabetes mellitus (GDM), which is characterised by the onset of hyperglycaemia during the second trimester of pregnancy, affects over 20% of pregnancies globally. Women diagnosed with GDM are at increased risk of developing short-term complications, including macrosomia, stillbirth, and neonatal metabolic disorders, and long-term outcomes such as cardiovascular disease and diabetes. Pregnancy induces physiological changes in the placenta and metabolic hormones, which can lead to increased insulin resistance. In certain instances, these changes may reveal pre-existing issues with insulin secretion. Defects in insulin secretion can arise from one or more of the following processes: (1) pre-existing pancreatic islet dysfunction caused by impaired pro-insulin synthesis, altered glucose sensing, or defective exocytosis of granules of insulin [1], or (2) insulin secretory defects from impaired incretin secretion or glucagon suppression [2]. On the other hand, insulin resistance can be due to (1) excess adiposity [3] contributed to by sedentary activities or dietary changes, (2) inflammation due to pre-pregnancy obesity, excessive postpartum weight retention [4], or hepatic insulin resistance [5].

Medical nutrition therapy (MNT) is an important primary intervention in 30% to 90% of women with GDM [6,7]. The aim of MNT is to achieve euglycaemia through tailored calorie and nutrient intake while ensuring the demands of a healthy pregnancy are met [6,8]. A meta-analysis of randomised controlled trials demonstrated that MNT combined with physical activity significantly improves glycaemic control during pregnancy and reduces the risk of macrosomia and neonatal adiposity [9]. Furthermore, women receiving appropriate advice on diet and physical activity are more likely to meet postpartum weight goals one year after delivery [9]. When MNT alone fails to achieve normoglycaemia, adjunct therapies such as insulin or metformin may be introduced alongside physical activity [8]. Effective MNT strategies must account for individual variations in glucose tolerance, self-monitored blood glucose values, pre-existing body weight, and gestational weight gain without inducing ketonaemia or impairing foetal growth [10]. Physical activity increases skeletal muscle glucose uptake, improves utero-placental oxidation capacity and complements MNT by preventing excessive weight gain [11,12,13].

Despite the established role of MNT in the management of GDM, implementation of MNT in clinical practice remains controversial. The challenge of MNT lies in achieving a balance between meeting the nutritional demands of pregnancy and ensuring effective blood glucose control to reduce the risk of macrosomia while simultaneously preventing starvation ketosis [14]. Current guidelines emphasise that MNT should be delivered by a registered dietitian or a healthcare professional with specialised expertise in GDM [8]. In this review, we aim: (1) to summarise the current evidence on MNT interventions for women with gestational diabetes mellitus (GDM), (2) to identify specific gaps in MNT management for this population, and (3) to highlight key research priorities that can advance clinical practice and improve maternal and foetal outcomes. By addressing these aims, this review seeks to provide a clearer direction for future research and clinical interventions in GDM care.

## 2. Search Strategy and Selection Criteria

A literature search was conducted to identify studies on MNT in women with GDM, published up to January 2025. Databases including PubMed database, Scopus database, Ovid Medline, Ovid Embase were searched for English-language articles. We excluded animal and cell studies and only included prospective and retrospective observational cohort studies, randomised controlled trials, reviews, cross-sectional studies, and best-practice guidelines. Search terms used included different combinations of the following: ‘nutrition requirements’ AND ‘women with gestational diabetes’ OR ‘macronutrient and micronutrient requirements’ AND ‘gestational diabetes’ OR ‘energy status and requirements’ AND ‘women with gestational diabetes.’ Nutrition plans for women with GDM are typically individualised to ensure appropriate calorie intake for mother and foetus without incurring excessive maternal gestational weight gain or maternal ketonaemia; thus, we also used search terms such as ‘gestational weight gain’ OR ‘calorie requirements’ OR ‘calorie intake’ OR ‘maternal ketonemia’ AND ‘women with gestational diabetes.’ In terms of the specific requirements needed during pregnancy for women with GDM, we used more specific terminologies such as ‘carbohydrate requirements’ AND ‘women with gestational diabetes,’ ‘type of carbohydrates’ OR ‘glycemic index of carbohydrates in women with gestational diabetes,’ ‘quality of carbohydrates needed during gestational diabetes,’ ‘carbohydrate restriction during gestational diabetes,’ ‘protein requirements,’ ‘fat requirements,’ ‘vitamins and mineral requirements’ AND ‘gestational diabetes.’ In terms of cultural particularities in women with GDM, we used search terms such as ‘cultural beliefs’ OR ‘perspectives AND understanding of medical nutrition therapy’ OR ‘dietary practices’ OR ‘dietary habits’ OR ‘insights to MNT’ OR ‘barriers to MNT’ AND ‘gestational diabetes’.

## 3. Nutritional Requirements in (Normal) Pregnancy

Human pregnancy is a physiologically demanding process that requires significant energy expenditure. On average, energy expenditure increases by approximately 375 kJ (89 kcal) in the first trimester, 1200 kJ (286 kcal) in the second trimester, and 1950 kJ (466 kcal) in the third trimester [15]. Throughout pregnancy, metabolic adaptations occur to ensure an adequate supply of nutrients for both the mother and the developing foetus.

In early pregnancy, glucose tolerance remains normal and basal hepatic glucose production is comparable to the non-pregnant state. However, insulin secretion in response to oral glucose increases by at least 120% in the first trimester [16,17,18]. As pregnancy progresses, basal glucose concentrations decrease by 0.56 to 0.83 mmol/L by the third trimester, while both first- and second-phase insulin secretion rise by up to 3.5-fold to accommodate the increase in insulin resistance [18,19,20]. These changes are partly mediated by alterations in hormonal milieu such as progesterone, prolactin, and human placental lactogen (hPL) levels [21,22]. In early pregnancy, hPL promotes maternal insulin resistance to prioritize glucose allocation to the foetus, while progesterone and oestrogen stimulate lipid deposition and fat storage to meet the energy requirements of late pregnancy and lactation. In later stages of pregnancy, elevated levels of hPL, prolactin, and glucagon promote lipolysis and fat mobilisation, shifting metabolic metabolism towards alternative energy sources while preserving plasma glucose and amino acid for foetal use. These adaptations are essential, as the foetus requires approximately 20–25 g obligatory glucose use per day in late gestation [23,24,25]. Women with GDM exhibit reduced suppression of hepatic glucose production and an exaggerated first-phase increase in insulin response compared to controls in late pregnancy. Elevated plasma insulin may inhibit fatty acid oxidation, and impaired triglyceride oxidation triglycerides may contribute to macrosomia [18,26]. Adequate caloric intake is therefore needed to balance maternal and foetal needs, maintain glycaemic control, and prevent excessive gestational weight gain (GWG).

Both under- and overnutrition during pregnancy can influence foetal size, body composition at birth, and long-term metabolic health [27]. Maternal undernutrition may lead to foetal growth restriction (FGR), characterised by reduced pancreatic growth, impaired insulin secretion, and hepatic insulin resistance [28,29,30]. When coupled with postnatal calorie excess, this FGR phenotype increases the risk of childhood and adult obesity, insulin resistance, and diabetes mellitus [31]. Conversely, foetal overnutrition can drive excessive insulin secretion and fat accretion, resulting in macrosomia [32].

## 4. Gestational Weight Gain (GWG) in Women with GDM

Gestational weight gain (GWG) is influenced by the developing foetus, maternal tissue changes (e.g., body fluids, amniotic fluids, extracellular fluid volume), and maternal adipose tissue expansion, including uterine and mammary gland growth [33,34]. The 2009 Institute of Medicine (IOM) guidelines recommend GWG ranges based on pre-pregnancy BMI of 12.5–18 kg for underweight (BMI < 18.5 kg/m^2^), 11.5–16 kg for normal (BMI 18–24.9 kg/m^2^), and 7–11.5 kg for overweight (BMI 25–29.9 kg/m^2^) women [34]. Excessive GWG is more common in women who are overweight or obese (64%) and is linked to higher risks of delivering large-for-gestational-age (LGA) infants across ethnicities (OR 1.68–1.93) [35]. Conversely, women who were underweight showed the highest prevalence of falling below the GWG guidelines (43%) [35].

Women with GDM tend to gain more weight before 24 weeks’ gestation compared to women without GDM (women with GDM: 6.7 kg vs. women without GDM: 5.1 kg) [36]. Excessive GWG in women with GDM increases risks of pre-eclampsia, caesarean section, macrosomia, and LGA (36%–83% higher risk per 0.5 kg per week increase in weight gain after diagnosis of GDM) [37]. Early GWG (0.27 to 0.40 kg/week) is associated with an elevated risk of GDM (OR 1.43–1.74) [38], driven by maternal fat accumulation [38] and reduced insulin sensitivity [39] in early pregnancy. Postpartum weight retention at 1 year post-delivery further increases the risk of glucose intolerance by 2.5-fold, independently of pre-pregnancy BMI (95% CI 1.46, 3.68) [40].

Excessive GWG in women with GDM worsens hyperglycaemia [41] and adverse pregnancy outcomes [42,43,44], although specific GWG targets remain undefined. Lower GWG in women with GDM [44,45,46,47,48,49] is associated with reduced risks of pregnancy-induced hypertension, pre-eclampsia [49,50,51], caesarean section [47], and macrosomia [43,49] without increasing small-for-gestational-age (SGA) risks [43,49]. A GWG of less than 5 kg in women with morbid obesity and GDM reduces the risk of macrosomia and hypertension without inducing pregnancy complications [52]. A retrospective cohort study in Australia also found that reducing the upper Institute of Medicine target by 2 kg for women with obesity or women with GDM did not affect pregnancy outcomes [47]. However, excessive weight loss may increase ketogenesis [53] and negatively impact foetal growth and future neurocognitive development [54]. Balancing GWG during pregnancy is critical to optimize maternal and foetal outcomes.

## 5. Caloric Recommendation in Women with GDM

During pregnancy, dietary energy must compensate for energy stored in maternal and foetal tissues and the increase in energy expenditure (EE) due to elevated basal metabolic rate (BMR) and changes in physical activity. In a healthy pregnancy, the BMR rises at an average rate of 10.7 ± 5.4 kcal/gestational week, with variations based on pre-pregnancy BMI: women with lower BMI (≤19.8 kg/m^2^) experience a lower BMR, at 8.8 ± 4.5 kcal/week, while those with higher BMI (≥26 kg/m^2^) have a higher BMR, at 16.3 ± 5.4 kcal/week. The increase in 24 h total energy expenditure (TEE) is primarily driven by an increase in BMR, with women with BMI of 19.8–26 kg/m^2^ experiencing an average total energy expenditure (TEE) increase of 7.4 ± 10.2 kcal/gestational week compared to a smaller TEE increase of 2.0 ± 15.1 and 2.9 ± 16.2 kcal/week in low- (≤19.8 kg/m^2^) and high-BMI groups (≥26 kg/m^2^), respectively. Activity-related energy expenditure declines as pregnancy progresses and is independent of GWG [55]. For a healthy pregnancy, women with normal BMI of 19.8–26 kg/m^2^ require a total energy requirement of 2182 kcal/day in the first trimester, 2561 kcal/day in the second trimester, and 2723 kcal/day in the third trimester. This translates to negligible additional energy needs in first trimester, but an extra 350 kcal/day in the second trimester and 500 kcal/day in the third trimester compared to non-pregnant values [56].

Guidelines for daily caloric intake in women with GDM vary widely [57]. The Endocrine Society recommends moderate energy restriction (1600 to 1800 kcal per day) or a 33% reduction in total calories for overweight/obese women with GDM to improve maternal glycaemia without compromising foetal growth or inducing ketosis [8]. In contrast, Diabetes Canada Clinical Practice Guidelines and the American Diabetes Association emphasize a food plan of at least 175 g/day of carbohydrates distributed across several meals, without specifying calorie limits [10,58]. The German Diabetes Association advises adjusting caloric intake based on pre-pregnancy BMI, restricting intake to a maximum of 24 kcal/kg/day, or reducing calories by 30%–33% in women with GDM and obesity (BMI ≥ 30 kg/m^2^) [59].

Emerging evidence suggests that modest energy restriction in women with GDM does not increase adverse outcomes or SGA rates compared to the baseline population [60,61,62]. Hodson K et al. [63] found that a 4-week 1200 kcal/day diet in women with GDM led to a 1.6 ± 1.7 kg weight loss and a 1.9% reduction in liver triacylglycerol levels compared to controls. Similarly, Kusinski LC et al. [64] demonstrated in a randomised controlled trial that a 1200 kcal/day diet in women with GDM and BMI ≥ 25 kg/m^2^ significantly reduced the need for long-acting insulin (OR 0.36, 95% CI 0.18–0.70) compared to a 2000 kcal/day diet, independently of maternal sociodemographic status, pre-pregnancy BMI, or age. Postnatal haemoglobin A1c (HbA1c) at 3 months was lower in the intervention group (5.5% vs. 5.8%), with no increase in neonatal intensive care unit (NICU) admissions, neonatal death, cord blood C-peptide measurement, or SGA infant rates. A 3 kg weight loss during later pregnancy improved maternal glucose levels, reduced postnatal HbA1c by 0.33%, and lowered the rates of LGA infants (OR 0.52, 95% CI 0.29, 0.93) without increasing SGA rates. These findings suggest that a 1200 kcal/day diet is safe for women with GDM and BMI ≥ 25 kg/m^2^. However, further research and collaborative efforts are needed to refine caloric requirements for women with GDM from diverse sociocultural backgrounds and integrate this evidence into clinical practice.

## 6. Carbohydrate Requirements: Amount, Type, and Distribution

Carbohydrates are important sources of energy for both mothers and foetuses. Dietary carbohydrates (e.g., white bread, potatoes) are readily digested and absorbed, and affect postprandial glucose levels. The foetus relies on glucose for growth and brain development, while the placenta utilises glucose for energy. Excessive glucose levels have been associated with increased infant size and adiposity up to 32-weeks of gestation [65]. Guidelines recommend a minimum carbohydrate intake of 175 g/day for pregnant women [10,58], based on an estimated average requirement of 100 g/day for non-pregnant women and an additional 33 g needed for foetal brain development, as well as an increase of 15% to account for population coefficient of variation [66].

Current evidence does not clearly define a safe lower limit of carbohydrate or caloric intake during pregnancy. Nonetheless, consumption of <42% of energy as carbohydrates has been shown to obviate the need for insulin therapy [67]. A key concern with carbohydrate restriction is the rise in maternal free fatty acids and ketone production, which can lead to pre-eclampsia [68], foetal demise [68], and neurodevelopmental complications [69]. In early pregnancy, maternal blood glucose levels typically decrease, and fat oxidation becomes the predominant energy source [70]. Carbohydrate restriction may alter the ratio of glucagon to insulin and promote free fatty acid conversion to beta-hydroxybutyrate (BOHB) [71]. Studies by Knopp RH et al. [72] showed that a 50% calorie restriction in women with GDM at 30–33 weeks gestation led to a twofold increase in ketonuria, but this was not seen with a 33% calorie-restricted diet (1600 kcal/day). Harreiter et al. [73] conducted a randomised controlled trial comparing women adhering to ~4–8 weeks of healthy eating with a reduced carbohydrate intake frequency at 24–28 weeks’ gestation to those on a normal diet. They found that women adhering to ~4–8 weeks of reduced carbohydrate intake at 24–28 weeks had higher BOHB levels (0.082 vs. 0.068 mmol/L; *p* < 0.05) and elevated fasting glucose (4.7 vs. 4.6 mmol/L; *p* < 0.05) compared to those on a normal diet, but these differences were not present at 35–37 weeks’ gestation [73]. Mijatovic J et al. [74] observed that a modestly lower carbohydrate intake of 165 g/day (~38–39% of energy intake) at 28.5 ± 0.4 weeks in women with GDM did not significantly raise ketone levels compared to those treated with 190 g of carbohydrates per day. However, the effects of a low-carbohydrate intake of <65 g/day are less clear, as most women could not achieve such low carbohydrate levels during pregnancy.

Major C [67] and Moreno-Castilla et al. [75] initiated carbohydrate restriction intervention in women with GDM within a week of diagnosis of GDM at 24 to 28 weeks’ gestation, whilst Wong et al. [76] included studies in their meta-analysis where calorie restriction began at gestation weeks 29 to 31 of pregnancy.

In terms of pregnancy outcomes, while early studies suggested that a low-carbohydrate diet reduced the insulin requirements and rates of LGA newborns [67], later studies found no significant benefits in terms of insulin use, timing of insulin initiation, or pregnancy outcomes in women with GDM compared to normal carbohydrate intake [74,75]. A meta-analysis supported these findings, showing no improvement in fasting blood glucose, insulin requirements, birth weight, caesarean delivery rates, macrosomia, or the incidence of LGA or SGA infants with a low-carbohydrate diet initiated at 29–31 weeks [76]. Rather unexpectedly, fasting glucose was reported to be paradoxically higher in women randomised to the relatively lower-carbohydrate (40% of total calories) and higher-fat diet than women consuming a relatively higher-carbohydrate diet (60% of total calories) [77]. Higher fasting glucose with low carbohydrate intake could be attributed to the increased release of free fatty acids in response to a lower-carbohydrate diet, leading to worsening insulin resistance [78].

Overall, robust evidence on dietary recommendations of carbohydrates for women with GDM remains limited, with many studies hindered by small samples, inconsistent outcomes, and varied gestational ages at initiation of caloric restriction interventions. However, pooled meta-analyses suggest that a modified carbohydrate-restricted diet may reduce birth weight and improve maternal glycaemic control without increasing blood ketone levels [79,80].

### Type of Carbohydrate: Low Glycaemic Index vs. High Glycaemic Index

The glycaemic index (GI), proposed by Jenkins et al. in 1981 [81], measures the rate and ability of certain foods to elevate blood glucose levels after meals compared to a standard quantity of carbohydrates. Based on the glucose response profiles, carbohydrates are classified as high GI (HGI) (e.g., soft drinks, desserts) or low GI (LGI) (e.g., cereals, nuts). Some early studies indicated that an HGI diet may be linked to increased foetoplacental growth and maternal weight gain, which can lead to foetal macrosomia. Conversely, an LGI diet may reduce insulin resistance during pregnancy, support normal infant birth weight, and help prevent excessive maternal weight gain [82,83,84]. However, findings from subsequent trials showed variable outcomes of LGI diets over conventional diets. A randomised controlled trial found that an LGI diet started during the second trimester produced similar birth outcomes and did not convey any difference in HbA1c, lipid, or insulin requirements in women at risk of GDM compared to those on a conventional diet [85]. The ROLO (Randomized Control Trial of Low Glycaemic Index Diet) study evaluated 800 women with a history of LGA infants and found that mothers on an LGI diet had less GWG (12.2 kg vs. 13.7 kg; mean difference −1.3 kg, 95% confidence interval −2.4 to −0.2; *p* = 0.01) and less glucose intolerance compared to those receiving a standard diet, but the incidence of LGA babies was not influenced by the intervention diet [86]. Further analysis of 621 participants in the ROLO group found that mothers on an LGI diet had lower circulating insulin from 0 to 28 weeks’ gestation, but foetal and maternal adipokines and inflammatory markers remained unchanged [87]. Also, the ROLO study group found a positive association between neonatal central adiposity and maternal dietary fat intake and postprandial glucose at 28 weeks’ gestation [88]. A comprehensive meta-analysis of 11 trials involving 1985 women found that LGI diets significantly reduced fasting and postprandial glucose levels and the percentage of LGA compared to the control group. The effects of LGI diet on baby birth weight and GWG were heterogenous, and this variability likely stemmed from differences in participant characteristics, control treatments, and diagnostic criteria for GDM [89]. Despite these, the overall findings suggest that an LGI diet may improve several maternal metabolic outcomes without adverse effects on the infant [86,89].

Carbohydrates are not the only dietary component that can interfere with maternal glucose excursion and insulin signalling. Protein and fat intake have also been shown to potentially influence the risk of GDM. A systematic review of observational studies found that consumption of cholesterol ≥ 300 mg/day), red and processed meat (increment of 1 serving/day), and eggs (≥7 per week) were associated with a higher risk of GDM [90]. Another large prospective population-based study in China found that women who adhered to a diet high in wheat, rice, and fruit had lower odds of GDM (OR 0.54, 95% CI 0.26, 0.83), whereas women who adhered to higher protein intake (diet high in fish, meat, and eggs) had 1.83-fold the odds (95% CI 1.21, 2.79) of developing GDM [91]. Viguiliouk E et al. [92] also found that a median dose of 56 g/day of tree nuts, a low-GI food, was associated with improved fasting glucose in individuals with diabetes. The Australian Longitudinal Study on Women’s Health, which enrolled 3607 women and followed them for over 10 years, found that a relatively low-carbohydrate, high-fat, and high-protein diet was associated with an increased risk of GDM, while a higher intake of dietary fibre, fruit, and fruit juice decreased the risk of GDM [93]. Therefore, it is essential to consider the quantity of carbohydrates and their glycaemic index, the source of carbohydrates, and the associated composition of proteins and fats when prescribing MNT.

## 7. Protein Requirements in Women with GDM

During pregnancy, protein requirements increase to support protein deposition in foetal, placental, and maternal tissues, with an estimated 21 g/day needed during the second and third trimesters [66]. However, the source of dietary protein can influence glucose metabolism and the risk of GDM. Evidence from the Nurses’ Health Study II (1991–2001) showed that pre-pregnancy consumption of animal proteins, such as red meat, was associated with a 1.48-fold increased risk of GDM. In contrast, consumption of vegetable protein, especially nuts, was associated with a lower risk of GDM [94]. This association persists even after adjustment of dietary cholesterol and saturated fat intake. Furthermore, replacing 5% energy from animal protein with vegetable protein was associated with a 51% reduction in GDM risk (RR 0.49, 95% CI 0.29–0.84) [94]. Other studies have shown similar findings. For example, a 6-week soy-based diet (35% animal protein, 35% soy protein, and 30% plant proteins) significantly improved glycaemic parameters, including fasting glucose, serum insulin level, insulin resistance, and lipid profile, compared to a control diet (0.8 g/kg protein, 70% animal, and 30% plant protein) in women with GDM [95]. In Asian populations, higher animal protein intake (OR 2.87, 95% CI 1.58, 5.20) and vegetable protein (OR 1.78, 95% CI 0.99–3.20) were associated with increased GDM risk. Notably, specific animal protein sources, such as seafood and dairy proteins, were also linked with a higher risk of GDM, with odds ratios of 2.17 (95% CI 1.26–3.72) and 1.87 (95% CI 1.11–3.15) [96]. In terms of foetal outcomes, a Danish study found that mothers with GDM who had a mean protein intake of 93 ± 15 g/day during pregnancy had a higher tendency to deliver babies with higher abdominal mass percentage compared to controls [97]. However, there was no association between maternal protein intake and fasting insulin or insulin resistance in the offspring [97]. Soy protein consumption in women with GDM also reduces the incidence of newborn hyperbilirubinaemia and hospitalisations [95]. Overall, prioritising plant-based proteins, such as nuts, legumes, and soy, over animal-based proteins, particularly red meat and processed meats, may help mitigate the risk of GDM and improve metabolic outcomes. The quality and type of protein, rather than just the quantity, are key determinants of GDM risk, and more work is needed to understand the effects of maternal protein intake on foetal outcomes.

## 8. Lipid/Fat Requirements in Women with GDM

Lipid metabolism changes throughout pregnancy. During the first two trimesters, hyperinsulinaemia stimulates lipid synthesis, and maternal adipose tissues develop a heightened sensitivity to insulin. This greater sensitivity to insulin, along with increased activity of lipoprotein lipase (LPL), leads to fat accumulation. However, as pregnancy progresses towards the third trimester, maternal adipose LPL activity declines and fat mobilisation increases [98]. The foetus begins synthesising fatty acids as early as 28-weeks’ gestation and will continue to accrete fatty acids, reaching its peak in the last 5 weeks of gestation [99]. Foetal fatty acid accretion rates increase in the order of linoleic acid (LA) followed by arachidonic acid (AA) and docosahexaenoic acid (DHA) [100]. At term, most of the LA, AA, and DHA is stored in the infants’ adipose tissues, with DHA being a predominant fatty acid in the cerebral cortex and retina [100]. The placenta expresses specific receptors that facilitate the transfer of fatty acids to the foetus. Of the fatty acids, the long-chain polyunsaturated fatty acid (LCPUFA) is particularly essential for the development of membrane structures and foetal growth [100]. However, the interactions between maternal fatty acids and their transfer to the foetus are modified in GDM. Therefore, both total fat intake and quality of fat intake are crucial factors to consider in GDM-complicated pregnancies.

Elevated circulating free fatty acid levels are associated with maternal insulin resistance and LGA infants at delivery [101]. Women with GDM exhibit lower proportions of AA and DHA in maternal umbilical arteries compared to controls, suggesting increased foetal utilisation of LCPUFA [102,103]. Cord blood omega-6 LCPUFA levels also inversely correlate with neonatal birth weight in GDM-complicated pregnancies, unlike in controls [103]. Women with GDM and BMI > 25 kg/m^2^ also show reduced levels of LCPUFAs and elevated short-chain fatty acids (SFAs) in the third trimester compared to women with impaired glucose tolerance and controls [104]. Lower levels of alpha-linolenic acid (an omega-3 fatty acid), AA, and DHA are observed in red blood cell membranes in women with GDM compared to women without GDM [105,106]. Insulin resistance is inversely correlated with AA and DHA levels in red blood cell membranes, indicating altered fatty acid metabolism as a potential contributor to insulin resistance in women with GDM.

Adipose tissue also plays a key role in maintaining glucose homeostasis by efficiently storing lipids and regulating adipokine secretion to prevent lipotoxicity [107]. Subcutaneous adipose tissue (SAT) is the primary lipid storage depot. However, its capacity to expand and to undergo adipogenesis varies in different individuals [108]. During a healthy pregnancy, SAT expands, reaching its peak towards the second trimester [109], but impaired SAT expansion and adipogenesis can lead to adipocyte dysfunction, hindering glucose uptake and exacerbating insulin resistance [109,110]. The resultant excessive fat accumulation in visceral adipose tissue contributes to metabolic complications such as type 2 diabetes, non-alcoholic fatty liver disease, and obesity [111]. A cross-sectional study by McElwain CJ et al. [112] demonstrated that women with GDM and a BMI ≥ 30 kg/m^2^ had significant adipocyte hypoplasia in omental visceral adipose tissue depots compared to women with GDM and a BMI ˂ 30 kg/m^2^. Visceral adipocyte tissue from women with GDM and obesity also showed impaired intracellular insulin signalling pathways, including reduced expression of insulin receptor substrate 2, which is essential for glucose metabolism. These findings suggest adipocyte dysfunction and inability of pre-adipocytes to undergo adipogenesis may contribute to GDM.

Dietary fat guidelines for omega-3 fatty acid intake during pregnancy are similar to those for the general population. The American Association of Clinical Endocrinologists, the American College of Endocrinology, and the Obesity Society recommend daily consumption of 1.4 g omega-3 fatty acids (in which a minimum of 200 mg/day DHA) and 13 g omega-6 fatty acids [113]. Studies that assessed dietary fat intake of pregnant women reported that women who developed GDM consumed less omega-3 and omega-6 PUFAs than women unaffected by GDM [104,114]. A meta-analysis of randomised controlled trials showed that omega-3 fatty acids (with vitamin D or E) lowered fasting glucose and insulin resistance among women with GDM compared to a control group [115]. Another prospective study showed that linoleic acid (omega 6) supplementation was associated with a lower risk of GDM [116]. However, a longitudinal study reported increased GDM risk with omega-6 consumption [117]. Analysis from six randomised controlled trials (331 participants) showed significant improvements with omega-3 supplementation in women with GDM compared to a placebo group: fasting glucose decreased by 0.25 mmol/L, fasting insulin by 17.13 pmol/L, and insulin resistance by 0.51. Lipid metabolism also improved, with triglycerides and very-low-density lipoprotein (VLDL) cholesterol decreasing by 0.18 mmol/L and 0.1 mmol/L, respectively, while HDL cholesterol increased by 0.06 mmol/L. The inflammatory marker C-reactive protein decreased by 0.68 mmol/L in the omega-3 group [118]. Further research is needed to determine if LCPUFA intake could prevent GDM development.

## 9. Micronutrient Requirements

Recommendations for daily micronutrient intake in pregnancy in current guidelines are based on recommended daily allowance (RDA) data by the IOM guidelines [119].

### 9.1. Vitamin A

Vitamin A is a fat-soluble vitamin that plays a key role in vision, growth, immune function, and antioxidant activities. Substantial placental transport of vitamin A occurs between the mother and foetus, and thus an estimated 10% increase in vitamin A intake is recommended [119]. Pregnant women are typically advised to consume 770 µg/day of vitamin A [120]. Low vitamin A levels can contribute to night blindness (which affects up to 7.8% of pregnant women globally) [121]. Furthermore, low vitamin A levels correlate with infant mortality and low birth weight. However, supplementation with vitamin A has not been found to mitigate low birth weight or newborn death [122]. Retinol is associated with teratogenic effects, and hence an upper limit of 8000 IU/day has been established due to concerns regarding the associated risk of craniofacial and cardiac birth defects [123].

Vitamin A has been suggested to induce expression of gluconeogenic enzymes and increase blood glucose levels via hepatic glucose production or impaired insulin signalling in skeletal muscles [124]. However, human studies on the relationship between vitamin A levels and GDM have been unclear. Some investigators observed that low retinol levels in early pregnancy are associated with an increased risk of insulin-treated GDM [125], while other researchers suggest higher vitamin A levels are positively associated with GDM [126]. It is also unclear if this association could relate to obesity, which is also a risk factor for GDM. A cohort study reported no relationship between vitamin A and GDM in pregnant women with a BMI > 30 kg/m^2^ [127]. A cross-sectional study in China [128] reported a reference range of 0.22–0.63 mg/L for vitamin A levels in pregnant women, with the highest vitamin A levels in the second trimester (0.42 mg/L), followed by those in the first (0.41 mg/L, *p* < 0.001) and third trimesters (0.37 mg/L, *p* < 0.001), but the study did not collect data on adverse maternal and foetal outcomes within the study population. A recent meta-analysis of 32 studies showed higher vitamin A levels in women with GDM compared to women without GDM in the first trimester, at 24 to 28 weeks and more than 28 weeks of pregnancy. However, the studies included in the analysis were heterogenous and varied in terms of vitamin A assays, timing of vitamin A measurements, BMI, and GDM diagnostic criteria [129]. Thus, the relevance and potential use of vitamin A as a biomarker in women with GDM remains to be validated in large-scale cohort studies with standardised measurement methods.

### 9.2. Vitamin D

Vitamin D, whether 25-hydroxycholecalciferol or 1,25-dihydroxycholecalciferol, is transported across the placenta to the foetus. Deficiency in vitamin D during pregnancy is linked to maternal and foetal calcium disorders such as neonatal hypocalcaemia, infant tooth enamel hypoplasia, and maternal osteomalacia [130]. However, the optimal level of vitamin D in pregnancy remains undefined. It is advised that 1000–2000 IU/day of vitamin D supplementation can be initiated if vitamin D deficiency is diagnosed during pregnancy [131]. Routine supplementation of vitamin D to prevent pre-eclampsia is not currently recommended [132].

Vitamin D deficiency may also be associated with an increased risk of GDM [133]. A systematic review and meta-analysis of 29 prospective and nested case–control studies revealed a U-shaped non-linear association between serum vitamin D levels and GDM risk [134]. The lowest risk of GDM was observed in women with serum vitamin D levels of 40 to 90 nmol/L, while those with levels < 20 ng/mL had a 26% greater risk of GDM (OR 1.26 (95% CI 1.13–1.41)) [134]. Veronica T Boyle et al. [135] further reported that women with vitamin D levels < 75 nmol/L had a 2.3-fold the odds of developing GDM in early pregnancy. In a review of 22 trials, Palacious C et al. [136] found that vitamin D supplementation reduced the relative risk of GDM by 51% (95% CI 0.27 to 0.97). Similarly, a meta-analysis of 19 randomised controlled trials by Wang MM et al. demonstrated that vitamin D supplementation in pregnant women lowered serum fasting plasma glucose, HOMA-IR, and insulin concentration in women with GDM [137]. The mechanisms through which vitamin D deficiency influences the risk of GDM are not fully understood, but may include insulin resistance and disruption of glucose homeostasis [138].

### 9.3. Folic Acid/Vitamin B

Folates are important water-soluble vitamins for DNA synthesis, amino acid metabolism, protein synthesis, cell multiplication, and tissue growth. During pregnancy, folate requirements increase to support cellular metabolism and formation of red blood cells for foetal growth [139]. Folate supplementation during pre-conception and in early pregnancy can prevent development of neural tube defects [140]. The recommended folate intake during pregnancy is 600 µg/day, and an additional daily supplement of 400 µday/day is recommended for all women of childbearing age before conception [140]. Higher folate doses (4 to 5 mg/day) are recommended for high-risk women (e.g., women with neural tube defects, diabetes, anticonvulsant medication use) [140].

However, excess folate supplementation without adequate vitamin B_12_ (cyanocobalamin) can disrupt carbon metabolism, posing health risks. Studies on the effect of folate and vitamin B_12_ on glucose metabolism and GDM have reported varied outcomes. Some studies have suggested that women who receive daily folate supplementation in the first trimester may have a paradoxical increased risk of GDM [141,142]. In the Growing Up in Singapore Towards Healthy Outcomes (GUSTO) study [143], pregnant women with elevated folate levels at 26–28 weeks’ gestation had 1.3-fold the odds of developing GDM. Conversely, higher vitamin B_12_ levels at 26–28 weeks’ gestation were associated with lower fasting glucose and reduced risk of GDM. Similarly, Lai et al. found that women with combined vitamin B_12_ insufficiency and high folate levels had higher odds of developing GDM compared to those with normal vitamin B_12_ and high folate concentrations [144]. A prospective cohort study in Shanghai found the relationship between red blood cell folate and GDM to be nonlinear. Women with higher red blood cell folate ≥ 600 ng/mL at 9 to 13 weeks’ gestation were associated with ∼1.60-fold the odds of developing GDM (aOR 1.58 (95% CI 1.03–2.41); *p* = 0.033) compared with women who had RBC folate levels < 400 ng/mL [145]. Conversely, unlike previous studies that showed a negative association between vitamin B_12_ and GDM [146,147], Chen et al. reported that higher vitamin B_12_ levels at 9 to 13 weeks’ gestation were associated with a ~1.14-fold risk of GDM [145].

Folate and vitamin B_12_ are involved in nucleic acid synthesis, and their imbalance could disrupt carbon metabolism and the subsequent conversion of homocysteine to methionine, impairing nucleic acid synthesis. Further research is clearly needed to determine the levels of folate and vitamin B_12_ needed in early and mid-pregnancy for optimal maternal and offspring health.

## 10. Other Issues That Could Lead to Inadequate or Excess Nutrition Intake

### 10.1. Hyperemesis Gravidarum

Hyperemesis gravidarum is a severe form of nausea and vomiting affecting 0.3% to 10.8% of pregnancies [148]. Poor oral intake and fluid losses from recurrent emesis are often associated with electrolyte derangements, malnutrition, foetal growth restriction, low birth weight, and micronutrient deficiencies [149]. Women with hyperemesis are also at risk of inadequate carbohydrate intake, leading to ketonaemia, which influences embryogenesis and foetal development [150]. Moreover, inadequate fluid intake in hyperemesis can lead to dehydration, which is associated with a reduction in amniotic fluid volume, leading to complications of foetal deformities, umbilical cord compression, and death [151]. Severe vomiting and poor intake in hyperemesis cause rapid depletion of thiamine, with an increased risk of Wernicke’s encephalopathy [152]. Nutrition interventions should focus on fluid resuscitation and electrolyte stabilisation, followed by nutritional repletion and management of symptoms, with the aim of supporting adequate foetal growth [153]. More advanced nutrition support such as enteral nutrition and parenteral nutrition may be required in patients with severe symptoms [153].

### 10.2. Beliefs of Pregnant Women with GDM During Pregnancy

The first-line management of GDM requires substantial lifestyle changes, such as dietary adjustments, consistent blood glucose monitoring, and adherence to a self-care routine. However, encouraging women with GDM to engage in self-management can be especially challenging due to varying healthcare knowledge, sociocultural practices, attitudes, and beliefs related to food, which differ across population groups [154]. While globalisation has had an impact on food preferences, traditional dietary practices remain deeply ingrained, presenting a unique challenge for individuals with gestational diabetes. An individual’s food choices are influenced by well-established factors such as mood [155], cognitive process, religion, culture, climate, and socioeconomic conditions [156]. Other factors such as hunger, time pressure, and food cravings often impede adherence to medical nutrition therapy [157]. The challenge may be further intensified by feeling overwhelmed, not only due to the shock of the GDM diagnosis but also by the realisation of the necessity to minimize adverse pregnancy outcomes for the baby’s well-being [157]. Cultural beliefs further complicate dietary compliance: for example, some Latino Americans believe that abstaining from food during periods of craving might harm the baby [158]. Similarly, a study among South Asian immigrant women in Australia revealed that recommendations to reduce food intake were met with resistance, as these conflicted with cultural norms emphasising ‘weight gain’ and the notion of ‘eating for two’ [159]. Additionally, social obligations, such as consuming food prepared by family or friends, might hinder dietary adherence [159]. In northern England, women were found to prioritise advice from friends and family over medical recommendations, including unfounded beliefs such as avoiding nuts, pickles, and fish during early pregnancy to prevent miscarriage [160]. Buddhists may avoid certain vegetables for religious reasons [161], while prestige-seeking behaviours among wealthier individuals may lead to overconsumption and obesity [162,163]. Individuals in lower socioeconomic classes may rely on cheaper ultra-processed food rather than fresh or less processed food choices, potentially compromising their micronutrient intake [162,163]. In low- and middle-income countries (LMICs), cultural food practices often exacerbate challenges in the management of GDM [164,165]. For example, some women avoid eggs due to beliefs that they cause jaundice or hair loss, while others restrict protein-rich foods out of concern that they could lead to miscarriage. Milk consumption is sometimes avoided due to fears that it may cause vomiting or heartburn in the unborn child. These practices highlight the complexity of dietary self-management, which requires not only motivation but also an understanding of nutritional values within a limited time frame [157]. Rhoads-Baeza ME et al. [166] noted that low-income pregnant Hispanic women were reluctant to change their traditional consumption of fatty meats to healthier alternatives. Even if dietary changes were attempted, many expressed dissatisfaction about the restricted food options. Despite these challenges, most women remain committed to ensuring their baby’s well-being and are willing to adopt recommended dietary and glucose monitoring practices to ensure a healthy pregnancy [157]. Thus, effecting behavioural change would require strategies that deliver culturally appropriate educational resources tailored to the specific needs and health literacy levels of diverse population groups [157]. Such strategies must address deeply ingrained cultural beliefs and practices while providing practical, evidence-based guidance to support effective GDM nutrition management [167]. Investment in community-inclusive services that offer integrated antenatal and postnatal nutrition and diabetes education is necessary to address barriers stemming from misinformation and to ensure consistent messaging and standardised care practices. Pathways that incorporate nutrition education delivered by primary care providers could alleviate the burden on specialist clinicians and enable more efficient allocation of dietetic resources [168].

## 11. Future Directions

Despite much research on MNT in women with GDM, uncertainty still exists with regard to the optimal choice of therapy to reduce maternal cardiometabolic risks and prevent foetal macrosomia.

The World Health Organization (WHO) and various dietary guidelines in most parts of the world recommend consuming a diverse range of food groups to ensure adequate foetal growth [169,170]. However, promoting dietary diversity extends beyond increasing the variety of foods consumed. In western Ethiopia, adequate dietary diversity during pregnancy is associated with a lower risk of LGA infants, but factors such as wealth index, maternal care, women’s occupation, household food insecurity, gestational age, and stable meal frequency influence dietary diversity. Recommending food diversity is not possible unless multi-sectoral policies and collaborative programs are in place to strengthen women’s employment and promote maternal health education, food security, and nutrition equity [171]. Future nutrition intervention programs should be designed with consideration of the local needs and conditions of the country with an aim to improve maternal health [172].

Current published trials on nutrition intervention are relatively small compared to the high prevalence of diabetes in many regions. Additionally, most existing studies lack representation from Africans and Afro-Caribbean populations where diabetes prevalence is rising [173]. Studies aimed at stratifying participants by cultural dietary habits to assess differential nutrition responses will provide more detailed physiological information and facilitate precise nutrition counselling. However, studies in large, ethnically diverse populations will not be practically feasible unless there is sufficient funding and multi-sectoral collaboration from different countries. Therefore, significant work remains to establish the evidence base needed for a culturally adapted dietary recommendation.

Most dietary indices use records of 24 h food logs to generate scores for adherence to population-level dietary guidelines. However, these tools rarely account for social, environmental, or psychological perceptions of food choices Furthermore, dietary indices, such as the DASH score, are developed for high-income countries where food sources are plentiful and may not be applicable to lower-income countries [156,174]. Therefore, integration of contextual factors in nutrition assessment is essential to improve nutrition intervention. This could include application of a more holistic conceptual framework for dietary quality assessment [156], enabling nutritionists to better pinpoint areas for improvement and advise government agencies in developing future dietary guidelines.

While glucose tolerance tests and weight changes are often used in most trials, incorporating additional biomarkers such as inflammatory markers, oxidative stress indicators, and gut microbiota composition [175] could further enhance understanding of the metabolic responses to dietary interventions. However, identifying a biomarker that could reliably serve as an end-point in nutritional research is challenging. To date, there is a lack of established biomarkers that indicate a specific inflammatory pathway to differentiate between acute and chronic inflammation or predict chronic disease end-points. There is also a lack of reliable studies to ensure that these biomarkers are quantifiable and stable over time. Different assessment methods to measure these biomarkers via different kits with varying inter- and intra-assay coefficients of variability further influence the overall accuracy of the reported effect estimates [176,177]. Using multiple biomarkers may provide more useful insights when investigating the anti-inflammatory potential of a diet. However, this approach would require the development of a standardised assay to measure a panel of biomarkers representing a spectrum of immune-inflammatory pathways. Future work needs to validate combinations of biomarkers with disease outcome measures and to develop algorithms of diet patterns using specific biomarkers collected within a specific sampling period [177]. This would involve a large-scale intervention study with a strong multifactorial component to enable the direct comparison of various dietary patterns in relation to a range of biomarkers reflecting multiple inflammatory, oxidative, or immune-related pathways [178]. Conducting such a trial would require consensus and concerted efforts among practitioners and researchers in different parts of the world.

## 12. Conclusions

**Figure 1:**
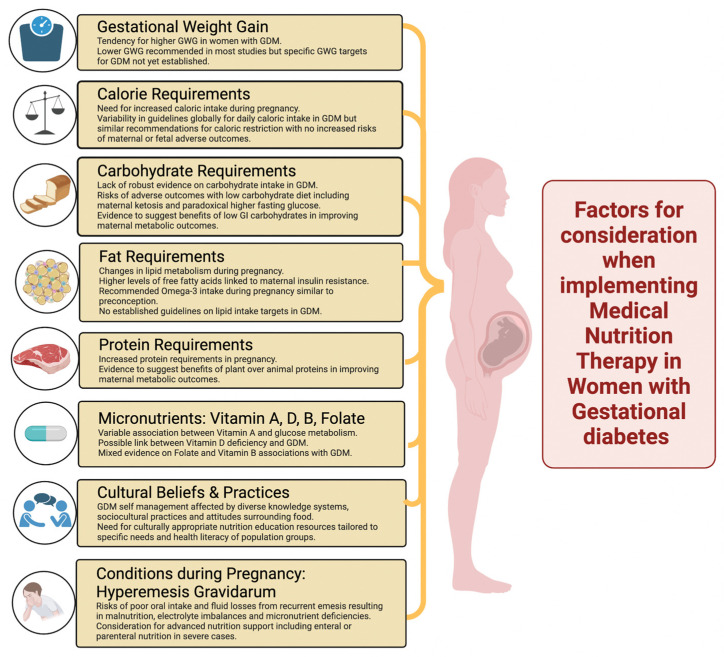
Factors for consideration in the implementation of medical nutrition therapy in women with gestational diabetes mellitus (GDM).

In conclusion, GDM is a complex condition influenced by physiological, hormonal, and metabolic changes during pregnancy, with significant implications for both maternal and foetal health. MNT remains the cornerstone of GDM management, but its implementation faces challenges due to cultural, socioeconomic, and psychological barriers, as well as variability in dietary practices and beliefs across different populations. Tailored nutritional strategies, including controlled carbohydrate intake, with emphasis on low-glycaemic-index foods and prioritisation of plant-based proteins and healthy fats (Figure 1), can optimize glycaemic control and reduce adverse outcomes. Addressing these challenges requires culturally sensitive education, consistent guidelines, and multidisciplinary care to ensure effective MNT delivery. Further research is needed to refine dietary recommendations, explore the role of micronutrients, and develop strategies to overcome barriers to ultimately improve maternal and foetal health outcomes in women with GDM.
